# Non-Intrusive Contact Respiratory Sensor for Vehicles

**DOI:** 10.3390/s22030880

**Published:** 2022-01-24

**Authors:** Quentin Meteier, Michiel Kindt, Leonardo Angelini, Omar Abou Khaled, Elena Mugellini

**Affiliations:** 1 HumanTech Institute, University of Applied Sciences and Arts of Western Switzerland//HES-SO, 1700 Fribourg, Switzerland; leonardo.angelini@hes-so.ch (L.A.); omar.aboukhaled@hes-so.ch (O.A.K.); elena.mugellini@hes-so.ch (E.M.); 2 University of Applied Sciences and Arts of Northwestern Switzerland//FHNW, 5210 Windisch, Switzerland; michiel.kindt@students.fhnw.ch

**Keywords:** contact, driver state, fusion, non-intrusive, respiration, sensor

## Abstract

In this work, we propose a low-cost solution capable of collecting the driver’s respiratory signal in a robust and non-intrusive way by contact with the chest and abdomen. It consists of a microcontroller and two piezoelectric sensors with their respective 3D printed plastic housings attached to the seat belt. An iterative process was conducted to find the optimal shape of the sensor housing. The location of the sensors can be easily adapted by sliding them along the seat belt. A few participants took part in three test sessions in a driving simulator. They had to perform various activities: resting, deep breathing, manual driving, and a non-driving-related task during automated driving. The subjects’ breathing rates were calculated from raw data collected with a reference chest belt, each sensor alone, and the fusion of the two. Results indicate that respiratory rate could be assessed from a single sensor located on the chest with an average absolute error of 0.92 min^−1^ across all periods, dropping to 0.13 min^−1^ during deep breathing. Sensor fusion did not improve system performance. A 4-pole filter with a cutoff frequency of 1 Hz emerged as the best option to minimize the error during the different periods. The results suggest that such a system could be used to assess the driver’s breathing rate while performing various activities in a vehicle.

## 1. Introduction

The driver’s condition can have a direct impact on his or her ability to operate the vehicle. The poor psychophysiological state of drivers is often the cause of car accidents. To address this problem, car manufacturers are increasing the level of automation in cars to support drivers. However, this raises other problems. Currently, partially automated cars are on the road (Level 2 automation as defined by the Society of Automotive Engineers (SAE; [1]). Soon, conditional automated driving (L3-SAE) could be adopted on public roads, depending on technological and legislative advances. If so, this type of vehicle will take over the dynamic driving task and ask drivers to regain control if necessary. However, for long periods without intervention, the driver may doze off or engage in other activities and not be in optimal conditions to take over control. Before cars are fully autonomous, it is therefore necessary to develop tools to best assist the driver. One way to do this is to collect data that continuously assesses the driver’s condition and to use this information to provide optimal assistance. This can be done by using the driver’s condition as input to an intelligent model that can adapt the information provided to the driver through the human-vehicle interfaces [2]. Physiological signals are one source of data providing intrinsic information about the driver’s condition. The relevance of these to assess the state of the driver according to various components such as fatigue and drowsiness [3,4,5,6,7,8], workload [8,9,10,11,12,13,13] or stress [8,14,15,16,17] has been proven in scientific literature.

In particular, the respiratory signal can be a valuable data source. The breathing pattern may change when drivers are drowsy [18], when they are talking, or when their cognitive load increases [12]. Measures of respiratory rate and respiratory variability can be calculated from the raw respiration signal. The latter can be recorded with reference laboratory respiratory sensors such as a respiratory belt transducer. Yet, it cannot be used in this context, although they have proven to be effective. If drivers are to be assessed for breathing under real-world driving conditions, it is not feasible to require them to wear a breathing belt every time they want to drive. Therefore, it is necessary to find a way to collect data in a robust, non-intrusive and continuous manner. Some contact-based systems were proposed in the literature, but either no quantitative analysis was made [19,20] or the performance (e.g., error rate) achieved was not reported [21,22]. Also, other solutions may be more expensive or more difficult to implement than the one proposed in this work [23]. This paper aims at filling the research gap by proposing a low-cost contact-based solution evaluated in user tests during simulated driving. This solution can further be implemented for driver monitoring in real conditions of both manual and automated driving.

## 2. Analysis

### 2.1. Respiration Rate Monitoring Methods

There are several ways to monitor an individual’s breathing pattern. These can be divided into two categories, namely, contact and non-contact methods [24]. Contact methods involve the use of devices or sensors attached to the user, and can be assessed by various means: breathed airflow, chest or abdominal movements related to breathing measured by strain wearable sensors [21], magnetic induction [19], the rate of carbon dioxide released during exhalation, the blood oxygen saturation level measured with an oximetry probe [25] or electrocardiogram (ECG)-derived respiratory rate [26]. The breathing signal can also be recorded using non-contact methods, through cameras [22], radars [27], speakers and microphones [28] or thermal imaging [18]. Both types of methods have their advantages and disadvantages. Contact methods involve the sensor or device being in contact with the driver. To record breathing in a car, methods involving the use of electrodes or an anemometer are not suitable. Overall, contact methods are all intrusive, to varying degrees. If such a method is used, the sensors and devices must be installed in places where there is constant contact with the driver, such as the seat and seat belt. In theory, non-contact methods are non-intrusive, which increases the chances of use and user acceptance. However, depending on the driver’s head position, posture, and movements, as well as the driving context, cameras or radar may not be accurate. Also, the noise of the vehicle and its environment might induce noise in the breathing signal when measuring breathing activity by microphone [28].

Previous researches have already developed systems to assess the breathing patterns of drivers using seat belts in the car. One of these systems used the principle of magnetic induction and showed promising results based on the shape of the collected signals. However, no quantitative evaluation was made for this system [19]. Another system estimated drivers’ respiratory rate from phase values sampled from several radio frequency identification (RFID) tags attached to the seat belt. A fairly low average error (between 0.10 and 0.15 bpm) could be achieved with this method. In a review of respiratory rate monitoring methods [24], the authors mention that chest and abdominal wall movements can be measured by contact-based methods, including impedance methods. Only one system used a piezoelectric sensor attached to the belt measuring the pressure generated by the abdominal region to collect the driver’s breathing signal [20]. They showed that a drowsy state can be observed visually and quantitatively with this system. However, the performance (e.g., error rate) achieved by the system is not reported. In this work, we propose to fill this gap by developing a contact-based system using a force-sensitive resistor (FSR) to measure thorax and abdomen movements through the seat belt. The system is compared to a reference belt by measuring the mean absolute error over several participants and several driving-related activities.

### 2.2. Sensor Selection

Developing a prototype with FSRs is a cost-effective solution that meets the requirements of portability, non-invasiveness and reliability. Several sensor references were analyzed such as the Tekscan A-201, the Ohmite FSR03CE and the Interlink FSR400. These sensors were selected based on our own experience and after having made a small state of the art of the existing sensors [29,30]. Important criteria were reliability through hysteresis (change in behavior during use), sensitivity (minimum force required to read data), force sensitive area and stability (defined here as “at rest” resistance). The cost-benefit analysis for selecting the most appropriate sensor is presented in Table 1. The Tekskan A-201 was selected for its reliability and sensitivity. Its low stability and small surface area will be compensated in the design of the sensor housing.

### 2.3. Signal Processing and Data Acquisition

The sensor data must be collected in a controlled and reproducible manner. Also, the prototype must assess the driver’s condition in real-time., the sampling rate must be sufficiently high, while the data and signal processing must be performed as quickly as possible. In addition, the cost-effectiveness and connectivity of the solution are of great importance. The system must robustly save the respiratory signals for later visualization and analysis. A comparison of different microcontrollers was performed on several criteria.

Possible alternatives/complementaries considered were the Arduino Uno, Raspberry Pi, Teensy 3.2, and Adafruit Metro boards. They were shortlisted based on mandatory and restrictive criteria such as ADC resolution, processing power, input pinout and price. The microprocessor requirements can be found in Table 2. The sampling rate must be up to 1 kHz to fairly compare with the respiratory signal collected with the reference chest belt (SS5LB, Biopac) since it was recorded at this sampling rate.

Based on these requirements, a cost-benefit analysis was performed to determine the most suitable solution(s) for the system, which is outlined in Table 3. The Adafruit’s Metro M4 was selected for its ADC resolution (12-bit ADC), high processing power (32-bit 120 MHz processor, 512 KB flash, 192 KB RAM), reasonable price, and additional features for future enhancements such as possible CircuitPython integration or built-in Bluetooth Low-Energy and WiFi capabilities (for the AirLift model).

## 3. Conception and Implementation

### 3.1. System Architecture

**Figure 1:**
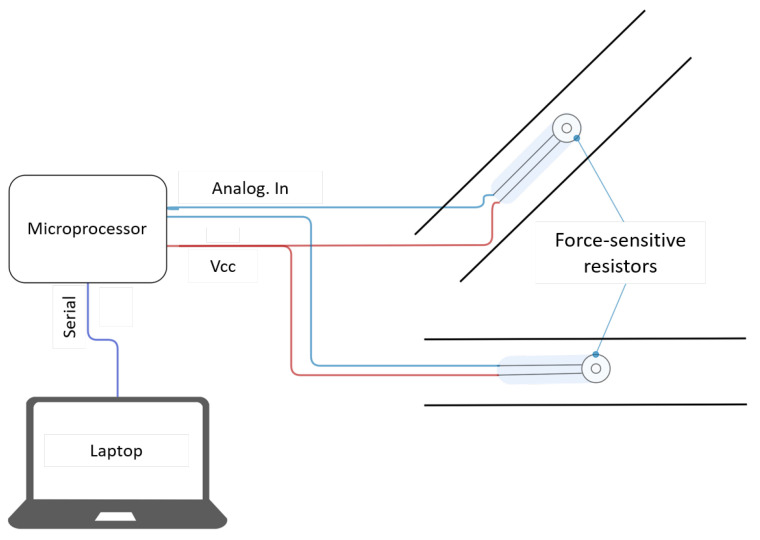
Illustration of the system architecture.

Figure 1 shows an overview of the system architecture. Two FSRs were attached to the seat belt through the housings. Initially, one sensor was to be placed on the abdomen and one on the chest. However, the designed system allowed the sensors to slide along the seat belt. Hence, different sensor locations were tested. The sensors were connected to a microprocessor responsible for collecting the signals and smoothing them with a digital filter. The data was sent in real-time to a laptop computer via a serial connection. The design of the system’s parts is explained in the subsections below.

### 3.2. Sensor Housing Design

The sensor housing must be unobtrusive, reliable, concentrate chest (or abdominal) pressure on the sensor surface, and be adjustable. The design process was iterative to find the optimal shape that met all these requirements. It led to the consideration of three different designs: a “U” shape, a concave shape, and a round shape. The parts were designed in Creo Parametric 6.0, and cut in Ultimaker’s Cura software. The parts were printed in polylactic acid (PLA) using the Ultimaker 2+ printer and a 0.4 mm nozzle.

**Figure 2:**
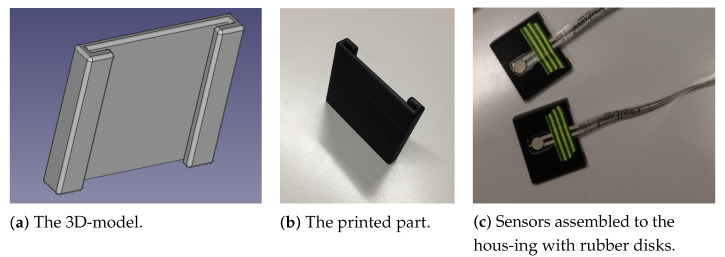
The 3D-model, the printed model and the assembly of the “U” shape.

The first housing design was the “U” shape. The mechanical part could slide along the seat belt. The FSR was wedged between the belt and the part and attached to the latter. Pressure from the chest/abdomen was transferred to the sensor and captured by the system. Figure 2a,b show the computer-designed prototype and the printed prototype of the “U” shape of the sensor housing, respectively. The sensors were assembled on the housing with small (5 mm diameter) rubber disks glued to them (see Figure 2c). However, this design provided a small contact area with the driver, but lacked robustness under motion, and exposed the sensors to external disturbances.

**Figure 3:**
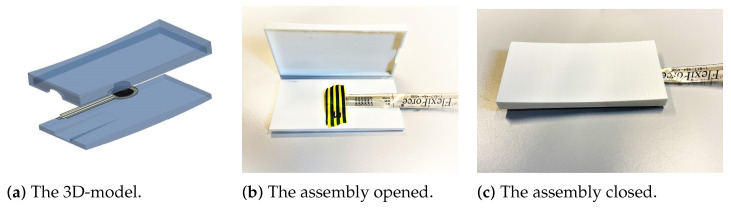
The 3D-model and the printed model of the concave shape, assembled with the sensors.

The second housing design had a concave shape. The objective of this design was to increase the contact area with the driver’s body. Figure 3a shows the computer-aided design (CAD) of the housing. The mold took a concave shape, consisting of two individual parts. The width was 5 centimeters, slightly larger than the international standards for seat belt width (46–49 mm). The length was 10 centimeters, for a total area of 50 cm^2^ on the driver’s chest. A radius of 580° ensured sufficient comfort and ease of positioning. A 15 cm wide inclined extrusion in the median plane of the bottom plate allows the sensor wires to exit the housing without being obstructed. Figure 3b,c show the printed concave housing assembled at the FSR with tape.

**Figure 4:**
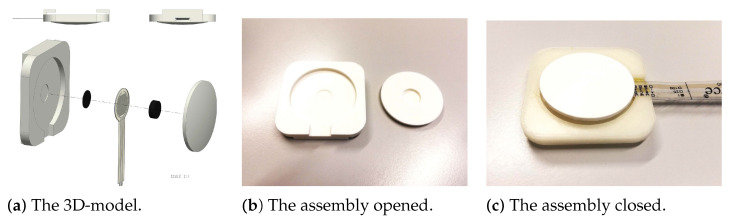
The 3D-model and the printed model of the round shape, assembled with the sensors.

The third housing design had a round shape. This new shape was designed because of the disadvantages of the previous model during testing. The shape and size of the concave mold did not perform as expected (see Section 4.1 and Section 4.2.1). A smaller housing size was also required because it physically interfered with the chest strap. Figure 4a shows the CAD of the round housing. It also consists of two parts, but the attachment mechanism has been simplified. This new design allows for better positional adaptability. The width and length were identical (5.2 cm), which is slightly larger than the seat belt. It was possible to easily slide the housing along the seat belt by positioning the belt between the upper surface of the housing and the L-shaped handles (see the upper left view in Figure 4a). This smaller profile allows both housings to be positioned above or below the reference sensor located in the middle of the subject’s chest. A 3 mm deep circular extrusion was made inside the upper housing. This cylindrical hole guides the translation of a convex disc that is in contact with the subject, under the seat belt. There are two silicone discs between the lower and upper parts of the housing, with the FSR wedged between them. They are held together by a viscous contact adhesive, which allows for movement and elasticity of the connection. On the side of the bottom part, an extrusion was made to allow the sensor to exit the housing without impeding the movement of the disc. The edges of the square shape were rounded with a radius of 10 mm so as not to interfere with the sensor as it moves in the seat. Figure 4b,c show the printed round housing assembled to the FSR with tape.

### 3.3. Electrical Circuitry

**Figure 5:**
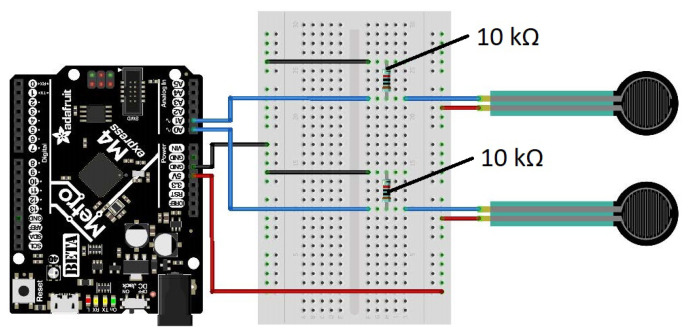
Sketch of the electrical wiring of the prototype.

The electrical circuitry of the system is shown in Figure 5. The two FSRs were wired on a breadboard and were powered by the microcontroller at 3.3 volts. The output of the sensors was fed into a resistor pulled to the ground. This voltage divider system was wired to the analog inputs of the microcontroller, where the signal can be sampled by the analog converters. For testing purposes, a button was added to start the microprocessor program. It also allowed for faster data analysis by setting the timestamp to 0 when starting the simulation.

### 3.4. Data Acquisition, Signal Processing and Data Transmission

The microcontroller sampled two sensors at a frequency of 100 Hz, filtered the data, and sent it serially. The sensor data were read by 2 ADC units in the Arduino Metro M4 microcontroller. Initially, the desired sampling rate was 1000 Hz to process the collected signal simultaneously with other physiological signals collected with the reference system (BioPac MP36). For performance reasons, it was lowered to 100 Hz, which is sufficiently high. BioPac recommends using a sampling rate of 50 Hz for proper analysis of respiratory signals. Thus, the choice of 100 Hz allowed for a margin of error while still having a properly recorded signal.

To smooth the respiratory signals before transmission, a transfer function for a low-pass exponential smoothing filter (RC filter) at the desired sampling rate (100 Hz) was created using Matlab. The cutoff frequency was set to 2 Hz. The transfer function was then discretized to obtain a discrete-time transfer function. It was applied to the two analog values collected by the sensors. Thus, the signal noise was filtered out above 2 Hz in real-time.

For the serial transmission, the baud rate (in bits per second) was set to 230,400 for fast data transmission. In addition, the packets should be as small as possible. The serial transmission was done through an 8-byte frame, containing a long variable for the time, and two integer values for the sensor readings. The variables must be of the same type (uint32_t in this case), which size the data frame to 12 bytes. Therefore, the ADC resolution was set to 12 bits, defining the range of respiration values from 0 to 4095. The column headings (Sensor 1, Sensor 2, Time) were sent through the serial port when the simulation started. Data was written as ASCII characters on the serial bus in the main loop.

### 3.5. Data Reception, Visualization and Storage

To visualize the signals in real-time to ensure the correct positioning of the sensors, the open-source serial logger SerialPlot was used. It allows for efficient data plotting and transmitting incoming data in a CSV file. The software recognizes active serial ports and simulation parameters can be recorded (baud rate and others). It facilitated system testing, especially in terms of speed and reproducibility. For each test session and participant, a separate CSV file was created containing the user’s breathing signals with time stamps. These files were saved on the laptop for subsequent calculation of indicators of respiratory rate variability.

### 3.6. Routine for the Calculation of Respiratory Rate Variability Indicators

An automatic routine was implemented in Python to calculate the participants’ respiratory rate from the signals collected during the test sessions. The signals collected by the two systems (the reference chest belt and the proposed system) were merged. Two additional signals were created from the signals captured with the proposed system: the average values of the two signals (sensor fusion), and the average value of the two signals after normalization between 0 and 1 (sensor fusion scaled). Using Neurokit [31], a Python module used for physiological data processing with advanced biosignal processing routines, the respiration rate of each participant was calculated, for each period (baseline, deep breathing, manual driving, automated driving) and each signal (sensor 1, sensor 2, reference sensor, sensor fusion, scaled sensor fusion). Then, the mean absolute error (MAE) of the signals were calculated for each sensor and each period, following the following formula (with N the number of subjects tested):

(1) $$MAE=\frac{1}{N}\sum_{i=1}^{N}\left|y_{i}-x_{i}\right|$$

## 4. Results

### 4.1. Experimental Procedure

**Figure 6:**
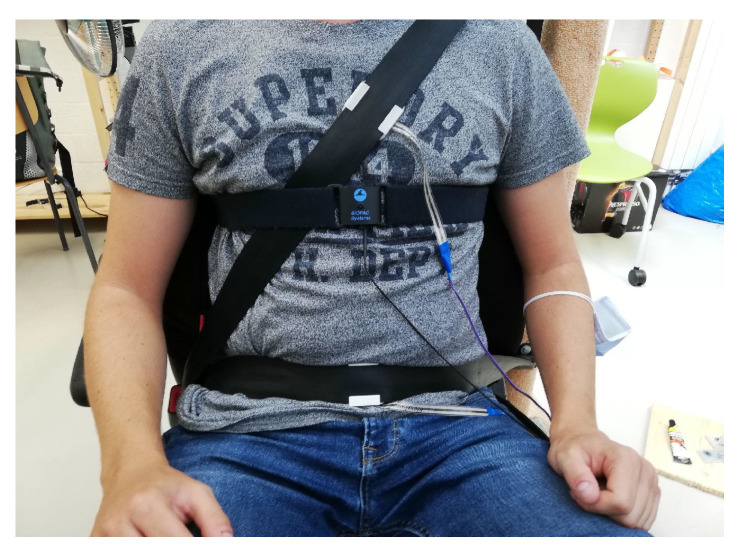
Setup for the second test session. The proposed system’s sensors were placed on the driver’s chest and abdomen, and the reference sensor attached at the chest.

Three test sessions were conducted to evaluate the different iterations of the sensor housing. Four subjects participated in the first session (session 1). The concave mold was used for both sensors. One was placed on the driver’s chest and the other on the driver’s abdomen. Six subjects participated in the second test session (session 2). The round-shaped sensor housings were used and placed identically on the seat belt (1 on the chest and 1 on the abdomen, see Figure 6). For the last test session (session 3), five participants were recruited. The round-shaped housings were also used, but this time the two sensors were placed on the driver’s chest. Both sensors were positioned symmetrically (between 5 and 10 cm) with respect to the breathing belt attached to the plexus.

All test sessions were conducted in the same fixed-base driving simulator. Subjects wore the gold standard chest belt (SS5LB, Biopac) and seat belt with the two FSR sensors attached. For each subject, the experiment consisted of four phases: a rest phase (baseline, 2 min), a deep breathing phase (1 min), a manual driving phase (2 min), and a conditional automated driving phase with the performance of a non-driving-related task (2 min). The Biopac student lab software collected the chest belt signal at a frequency of 1 kHz, whereas the proposed system’s data were collected at a sampling rate of 100 Hz on a laptop.

### 4.2. Evaluation

#### 4.2.1. Session 1

The first testing session revealed two major flaws in the system: the sensor housings were too long and impractical to be used simultaneously with the reference chest belt, and the tightening mechanism was ineffective on seat belts. For these reasons, a second iteration of the sensor housing was designed and tested in the second testing session.

#### 4.2.2. Session 2

Some outliers were found in this second test session. The amplitude of the collected signals was too low for some recordings and, therefore, the Neurokit module could not detect the peaks correctly. Nevertheless, the mean absolute error of the respiration rate could be calculated and the results can be found in the Table 4. We see that poor results are obtained for the sensor placed on the driver’s abdomen. The data could have been sporadically corrupted if the subject was wearing too much clothing or if his anatomy did not operate the FSR correctly. For this reason, both sensors were positioned on the driver’s chest during the last test session to ensure signal redundancy and to obtain a lower error.

#### 4.2.3. Session 3

**Figure 7:**
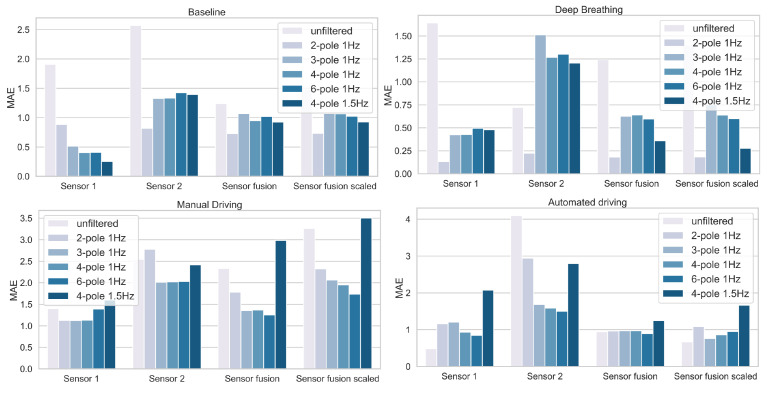
Effect of filter type and sensor on the mean absolute error of participants’ respiratory rate (in min^−1^), from data collected in the third test session. Filters varied in the number of poles and cut-off frequency.

Breathing data from five participants could be collected with the system in test session 3. The mean absolute error of the respiratory rate during the different periods of this test session is shown in Table 5. The results showed too much variability, suggesting excessive noise in the signal. For this reason, different filters were tested on the raw signals collected with the prototype. The objective was to find the most appropriate digital filter to minimize the error when calculating the respiration rate. Figure 7 shows the effect of filter type and sensor on the mean absolute error of the respiration rate, calculated from the data collected in session 3.

## 5. Discussion

The results of test sessions 2 and 3 suggest that breathing rate can be measured with an average error of about one breath per minute on average across all activities (resting, deep breathing, manual driving, and non-driving-related task during automated driving). The results show that the sensor should be placed on the chest to optimize the system’s performance. As mentioned earlier, the data from the sensor located on the driver’s abdomen could be noisy, either because the subjects were wearing too much clothing or because their anatomy did not operate the sensor properly. Even with two sensors located on the driver’s chest, the results obtained in test session 3 were no better than those obtained in test session 2. The convex disc that was in contact with the subject, under the seatbelt, stands out clearly from the mold and remains in good contact with the subject, despite the clothing or movements. Some movements of the chest may be absorbed by a too big thickness of clothing. A solution is to push the convex disc slightly harder on the subject automatically if the signal’s amplitude is too low, thanks to a spring system or an actuator. For further research, the influence of the number of clothing layers on the accuracy of contact-based systems should be investigated.

Sensor fusion was tested to improve system performance, but this was not the case. In test sessions 2 and 3, the signal from one of the two sensors contained noise, which affected the results of sensor fusion. More advanced sensor fusion techniques should be explored to improve the results. For example, an intelligent system would analyze the quality of the signal collected by the two sensors, before using the more qualitative signal to calculate the respiration rate.

To remove the noise in the signals and reduce the error rate, the effect of the digital filter type applied to the signals was tested. Focusing on the results obtained by sensor 1, signal filtering reduced the error except for the automated driving period (see Figure 7). The lowest mean absolute error (0.13 min^−1^) was obtained by applying a 2-pole filter with a cutoff frequency of 1 Hz to data recorded by a sensor located on the chest (sensor 1) during deep breathing. The 4-pole filter with a cutoff frequency of 1 Hz appeared to be the best option for minimizing the error in all periods, achieving an average absolute error of 0.5 min^−1^ during the resting and deep breathing periods, and 1 min^−1^ during the driving periods. During manual driving or a non-driving task, drivers may be moving. Their bodies may not be in contact with the belt, which may distort the assessment of their breathing rate. Since we have seen that a filter can reduce the error measured during certain activities but not necessarily for all, an intelligent system could recognize the task performed by the driver and select the corresponding filter, the one that would give the most accurate measurement of the breathing rate.

Even with the fusion of the sensors and the different filters tested on the signals, the results obtained are slightly worse than those of a previous study using RFID tags, which can estimate the driver’s breathing rate with a median error of 0.10 to 0.15 bpm [23]. The error increases with increasing respiratory rate. Another system that uses acoustic signals has also shown promising results (median error less than 0.35 breaths/min) [28]. However, the system can be disturbed if other vehicle occupants are talking. Also, the cost of these solution may be higher than the solution proposed in this work. Another non-contact based system using antenna achieved 0 to 8% of error, but it was evaluated only during 30 s on 3 test subjects [27]. Besides, results obtained in this work cannot be compared with some other systems, either because they did not perform a quantitative analysis [19,20], or because they statistically analyzed the results obtained by the proposed system with a reference system without reporting the error values in the manuscript [21,22].

Although the results obtained are encouraging, this work still has certain limitations. A larger sample of subjects would be necessary to obtain more consistent results. Indeed, with four to six subjects tested, the mean absolute error drastically increased when the sensor was not properly positioned for one subject. The results presented here provide an approximation of the performance that can be achieved by such an inexpensive contact solution to assess driver respiration rate. Another limitation concerns the reference chest belt. It was used as a reference value of breathing rate, but it could also be wrong and far from the actual breathing rate of drivers. Another reference measurement should be used to overcome this problem, using a non-contact method. Besides, no specific methodology was employed to choose the position of the sensors on the chest or the abdomen. It should be better defined to ensure that the sensor is positioned the same for all test subjects. Sensor’s placement could also have an influence on the system’s performance.

## 6. Conclusions

This paper proposes an innovative and low-cost solution to measure driver respiration rate by a contact-based method using two force-sensitive resistors attached to the seat belt by a specially designed housing. An iterative process was used to design and print the sensor housing. The housings were designed so that the position of the sensors could be easily adjusted by sliding along the belt. The system was evaluated with a few test subjects during different activities in a driving simulator: resting, deep breathing, manual driving, and non-driving activity during automated driving. The results indicate that breathing rate could be assessed from chest movement with an average absolute error of 0.92 min^−1^ across all periods. Sensor fusion did not improve the accuracy of the system. A 4-pole filter with a cutoff frequency of 1 Hz emerged as the best option for minimizing error in all periods, achieving an average absolute error of 0.5 min^−1^ during rest periods, and 1 min^−1^ during driving periods. The lowest mean absolute error (0.13 min^−1^) was achieved by applying a bipolar filter with a cutoff frequency of 1 Hz to data recorded by a sensor located on the chest (sensor 1) during deep breathing. 

## Figures and Tables

**Table 1:** Cost-benefit analysis for the choice of force-sensitive resistor (FSR). Weight: Importance of each criterion (out of a total of 100); Factor: Evaluation of the sensor’s ability to meet this criterion; Score: Score achieved by the sensor for a given criterion. Score = Weight ∗ Factor. Bold value indicates the best score.

Criteria	Weight	Tekscan A-201		Ohmite FSR03CE		Interlink FSR400	
		Factor	Score	Factor	Score	Factor	Score
Reliability	30	4	120	3	90	3	90
Sensitivity	40	5	200	3	120	3	120
Surface area	10	3	30	5	50	4	40
Stability	20	3	60	4	80	4	80
**Total**			**410**		340		330

**Table 2:** Requirements for the microprocessor. A high score in the Priority column means greater importance for that criterion. CP = Circuit Python; ADC = Analog-to-digital converter; Roles: M = Mandatory, R = Restriction, W = Wish, O = Optimization.

Object	Property	Measure	Role	Priority
Architecture	n-bit microprocessor	>=32-bit	W	5
ADC Resolution	Bit depth	>10-bit	R	15
Processing power	Clock speed	>=16 MHz	R	10
Input pinout	# analog pins	>=3	M	10
Board size	Board surface area	<Arduino	O	5
Price	Euros	<50	R	5
Support	Documentation	Y/N	M	10
Program memory	Flash memory size	>32 KB	W	5
CP	CP support	Y/N	W	10
Wifi + Bluetooth	Integrated	Y/N	W	10
Hardware connectivity	Jumper/soldering pins	Jumper	W	5

**Table 3:** Cost-benefit analysis for the microprocessor. Weight: Importance of each criterion (out of a total of 100); Factor (F): Evaluation of the sensor’s ability to meet this criterion; Score: Score achieved by the sensor for a given criterion. Score = Weight ∗ Factor. Bold value indicates the best score.

Criteria	Weight	Arduino Uno		Teensy 3.2		Metro M4		Raspberry Pi	
		F	Score	F	Score	F	Score	F	Score
Architecture	5	2	10	5	25	5	25	5	25
ADC Resolution	15	3	45	5	75	4	60	0	0
Processing power	10	2	20	3	30	4	40	5	50
Input pinout	10	5	50	5	50	5	50	5	50
Board size	5	3	15	5	25	3	15	3	15
Price	10	5	50	4	40	3	30	3	30
Support	10	5	50	2	20	4	40	5	50
Program memory	5	2	10	3	15	4	20	5	25
Circuit Python	10	0	0	0	0	5	5	5	50
Wifi + Bluetooth	10	0	0	0	0	5	50	5	50
Hardware	5	4	20	0	0	4	20	5	25
**Total**			270		280		**400**		370

**Table 4:** Mean absolute error of respiratory rate (in min^−1^) measured from data collected in the second pretest session, for each sensor and period. Values in bold are the lowest errors achieved by the system for each period and in average.

Period	Abdomen	Chest	Sensor Fusion	Sensor Fusion Scaled
Baseline	1.61	**0.32**	0.51	0.52
Deep breathing	2.24	1.25	**1.21**	**1.21**
Manual driving	3.56	**1.25**	2.08	2.39
Automated driving	2.98	**0.87**	1.30	1.21
Average	2.60	**0.92**	1.28	1.33

**Table 5:** Mean absolute error of respiratory rate (in min^−1^) measured from data collected in the third test session for each sensor and period. Values in bold are the lowest errors achieved by the system for each period and in average.

Period	Sensor 1	Sensor 2	Sensor Fusion	Sensor Fusion Scaled
Baseline	1.91	2.57	**1.24**	1.48
Deep breathing	1.64	**0.72**	1.24	1.40
Manual driving	**1.40**	2.54	2.34	3.26
Automated driving	**0.48**	4.10	0.95	0.67
Average	**1.36**	2.48	1.44	1.70

## Data Availability

Data can be retrieved upon request to the authors.

## Abbreviations

The following abbreviations are used in this manuscript:

ADC	Analog-to-digital converter
bpm	breaths per minute
CAD	Computer-Aided Design
ECG	Electrocardiogram
FSR	force sensitive resistor
Hz	Hertz
MAE	Mean Absolute Error
RFID	Radio Frequency IDentification
SAE	Society of Automotive Engineers
